# *trans*-Di­aqua­bis­(pyridazine-3-carboxyl­ato-κ^2^ *N* ^2^,*O*)copper(II)

**DOI:** 10.1107/S1600536814004334

**Published:** 2014-02-28

**Authors:** Aroa Pache, Amaia Iturrospe, Leire San Felices, Santiago Reinoso, Juan M. Gutiérrez-Zorrilla

**Affiliations:** a Departamento de Química Inorgánica, Facultad de Ciencia y Tecnología, Universidad de País Vasco (UPV/EHU), PO Box 644, E-48080 Bilbao, Spain

## Abstract

In the title compound, [Cu(C_5_H_3_N_2_O_2_)_2_(H_2_O)_2_], the Cu^II^ ion, located on an inversion center, exhibits an octa­hedral coordination geometry. The equatorial plane is defined by two *trans*-related *N*,*O*-bidentate pyridazine-3-carboxyl­ate ligands and the axial positions are occupied by two water mol­ecules. In the crystal, mol­ecules are connected by O—H⋯O hydrogen bonds between the water mol­ecules and the noncoordinating carboxyl­ate O atoms, forming layers parallel to the *bc* plane. The layers are stacked along the *a* axis by further O—H⋯O hydrogen bonds between the water mol­ecules and the coordinating carboxyl­ate O atoms. Weak C—H⋯O hydrogen bonds are also observed between the pyridazine rings and the water mol­ecules and between the pyridazine rings and the non-coordinating carboxyl­ate O atoms.

## Related literature   

For the isotypic zinc complex, see: Gryz *et al.* (2004 ▶). For a related cobalt(II) complex which contains two non-coordin­ating water mol­ecules, see: Artetxe *et al.* (2013 ▶). 
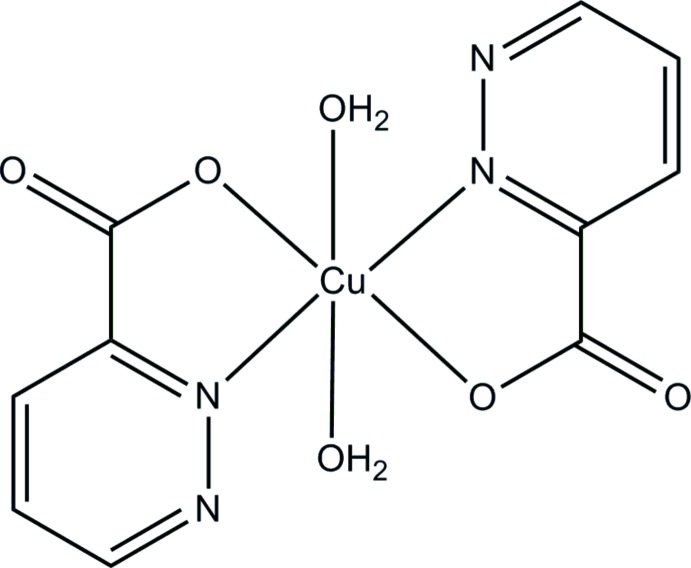


## Experimental   

### 

#### Crystal data  

[Cu(C_5_H_3_N_2_O_2_)_2_(H_2_O)_2_]
*M* *_r_* = 345.76
Monoclinic, 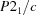

*a* = 5.4014 (1) Å
*b* = 11.5633 (3) Å
*c* = 9.6283 (2) Å
β = 101.837 (3)°
*V* = 588.58 (2) Å^3^
*Z* = 2
Mo *K*α radiation
μ = 1.89 mm^−1^
*T* = 100 K
0.19 × 0.09 × 0.06 mm

#### Data collection  

Agilent SuperNova diffractometer
Absorption correction: numerical (*CrysAlis PRO*; Agilent, 2012 ▶) *T*_min_ = 0.772, *T*_max_ = 0.898
2532 measured reflections
1216 independent reflections
1077 reflections with *I* > 2σ(*I*)
*R*_int_ = 0.022

#### Refinement  

*R*[*F* ^2^ > 2σ(*F* ^2^)] = 0.027
*wR*(*F* ^2^) = 0.065
*S* = 1.06
1216 reflections
105 parameters
3 restraints
H atoms treated by a mixture of independent and constrained refinement
Δρ_max_ = 0.44 e Å^−3^
Δρ_min_ = −0.45 e Å^−3^

### 

Data collection: *CrysAlis PRO* (Agilent, 2012 ▶); cell refinement: *CrysAlis PRO*; data reduction: *CrysAlis RED* (Agilent, 2012 ▶); program(s) used to solve structure: *OLEX2* (Dolomanov *et al.*, 2009 ▶); program(s) used to refine structure: *SHELXL97* (Sheldrick, 2008 ▶); molecular graphics: *ORTEP-3 for Windows* (Farrugia, 2012 ▶) and *DIAMOND* (Brandenburg, 2010 ▶); software used to prepare material for publication: *WinGX* (Farrugia, 2012 ▶).

## Supplementary Material

Crystal structure: contains datablock(s) I, global. DOI: 10.1107/S1600536814004334/is5342sup1.cif

Structure factors: contains datablock(s) I. DOI: 10.1107/S1600536814004334/is5342Isup2.hkl

CCDC reference: 988680

Additional supporting information:  crystallographic information; 3D view; checkCIF report

## Figures and Tables

**Table 1:** Selected bond lengths (Å)

Cu1—O1	1.9792 (15)
Cu1—N2	1.9822 (18)
Cu1—O1*W*	2.4207 (16)

**Table 2:** Hydrogen-bond geometry (Å, °)

*D*—H⋯*A*	*D*—H	H⋯*A*	*D*⋯*A*	*D*—H⋯*A*
O1*W*—H1*WA*⋯O1^i^	0.87 (2)	1.99 (2)	2.865 (2)	175 (2)
O1*W*—H1*WB*⋯O2^ii^	0.87 (1)	2.03 (2)	2.878 (2)	165 (3)
C4—H4⋯O1*W* ^iii^	0.93	2.52	3.403 (3)	158
C6—H6⋯O2^iv^	0.93	2.39	3.141 (3)	138

Symmetry codes: (i) 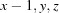; (ii) 
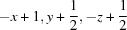
; (iii) 
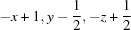
; (iv) 
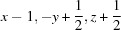
.

**Fig. 1.**
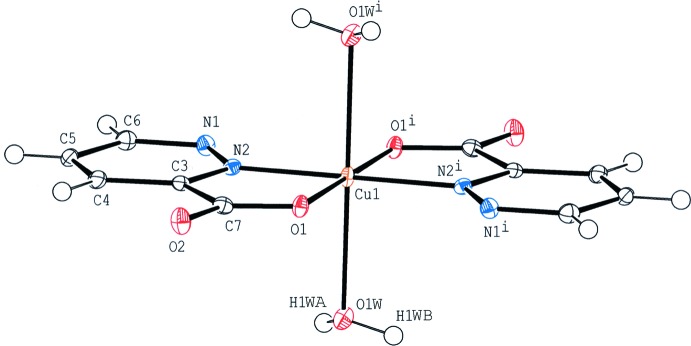
Molecular structure of the title compound, showing atom labelling and 50% probability displacement ellipsoids. [Symmetry code: (i) 1 - x, 1 - y, 1 - z.]

**Fig. 2.**
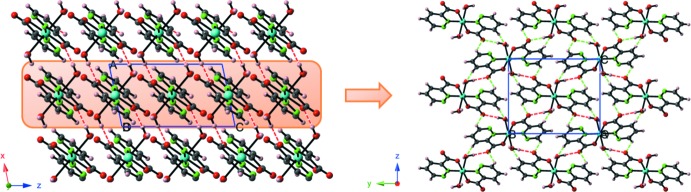
Left: View of the crystal packing along the b axis. Right: Projection of a layer along the a axis (O—H···O hydrogen bonds represented as dotted red lines and weak C—H···O interactions as dotted green lines).

## supplementary crystallographic information

### 1. Comment

The metal ion, the pyridazine ring and carboxylate atoms are coplanar. As expected, the Cu—O and Cu—N distances (Table 1) are similar to the Zn(II) and Co(II) analogue compounds (Gryz *et al.*, 2004; Artetxe *et al.*, 2013). Table 2 summarizes the geometrical parameters of the O—H···O and C—H···O hydrogen bonding interactions.

### 2. Experimental

To a solution of CuCl_2_.2H_2_O (34 mg, 0.2 mmol) in water (10 mL) 3-pyridazine carboxylic acid (48 mg, 0.4 mmol) was dropwise added and the resulting solution was stirred for 1 h at 80 °C. Blue prismatic crystals suitable for single-crystal X-ray diffraction were obtained by slow evaporation of the resulting solution after six days.

### 3. Refinement

H atoms of the water molecules were located in a Fourier difference map and refined isotropically with O—H bond lengths restrained to 0.88 (1) Å. All H atoms of the pyridazine ring were positioned geometrically and refined using a riding model with standard *SHELXL* parameters.

### Crystal data

**Table:**

[Cu(C_5_H_3_N_2_O_2_)_2_(H_2_O)_2_]	*F*(000) = 350
*M**_r_* = 345.76	*D*_x_ = 1.951 Mg m^−^^3^
Monoclinic, *P*2_1_/*c*	Mo *K*α radiation, λ = 0.71073 Å
Hall symbol: -P 2ybc	Cell parameters from 1663 reflections
*a* = 5.4014 (1) Å	θ = 2.8–28.4°
*b* = 11.5633 (3) Å	µ = 1.89 mm^−^^1^
*c* = 9.6283 (2) Å	*T* = 100 K
β = 101.837 (3)°	Prism, blue
*V* = 588.58 (2) Å^3^	0.19 × 0.09 × 0.06 mm
*Z* = 2	

### Data collection

**Table:**

Agilent SuperNova diffractometer	1216 independent reflections
Radiation source: Nova (Mo) X-ray micro-source	1077 reflections with *I* > 2σ(*I*)
Multilayer optics monochromator	*R*_int_ = 0.022
Detector resolution: 16.2439 pixels mm^-1^	θ_max_ = 26.5°, θ_min_ = 2.8°
ω scans	*h* = −6→6
Absorption correction: numerical (*CrysAlis PRO*; Agilent, 2012)	*k* = −13→14
*T*_min_ = 0.772, *T*_max_ = 0.898	*l* = −12→9
2532 measured reflections	

### Refinement

**Table:**

Refinement on *F*^2^	Primary atom site location: structure-invariant direct methods
Least-squares matrix: full	Secondary atom site location: difference Fourier map
*R*[*F*^2^ > 2σ(*F*^2^)] = 0.027	Hydrogen site location: inferred from neighbouring sites
*wR*(*F*^2^) = 0.065	H atoms treated by a mixture of independent and constrained refinement
*S* = 1.06	*w* = 1/[σ^2^(*F*_o_^2^) + (0.0235*P*)^2^ + 0.6643*P*] where *P* = (*F*_o_^2^ + 2*F*_c_^2^)/3
1216 reflections	(Δ/σ)_max_ < 0.001
105 parameters	Δρ_max_ = 0.44 e Å^−^^3^
3 restraints	Δρ_min_ = −0.45 e Å^−^^3^

### Special details

**Table:**

Experimental. IR (cm^-1^): 3554(s), 3315(s), 3233(s), 1628(s), 1571(m), 1578(s), 1365(w), 1231(w), 1152(w), 1091(w), 1072(w), 1039(w), 978(m), 851(m), 785(m), 722(m), 669(w), 536(w), 440(w).
Geometry. All s.u.'s (except the s.u. in the dihedral angle between two l.s. planes) are estimated using the full covariance matrix. The cell s.u.'s are taken into account individually in the estimation of s.u.'s in distances, angles and torsion angles; correlations between s.u.'s in cell parameters are only used when they are defined by crystal symmetry. An approximate (isotropic) treatment of cell s.u.'s is used for estimating s.u.'s involving l.s. planes.
Refinement. Refinement of *F*^2^ against ALL reflections. The weighted *R*-factor *wR* and goodness of fit *S* are based on *F*^2^, conventional *R*-factors *R* are based on *F*, with *F* set to zero for negative *F*^2^. The threshold expression of *F*^2^ > 2σ(*F*^2^) is used only for calculating *R*-factors(gt) *etc*. and is not relevant to the choice of reflections for refinement. *R*-factors based on *F*^2^ are statistically about twice as large as those based on *F*, and *R*- factors based on ALL data will be even larger.

### Fractional atomic coordinates and isotropic or equivalent isotropic displacement parameters (Å^2^)

**Table:**

	*x*	*y*	*z*	*U*_iso_*/*U*_eq_	
C3	0.4961 (4)	0.27046 (19)	0.4096 (2)	0.0083 (4)	
C4	0.4421 (4)	0.15278 (19)	0.3920 (2)	0.0107 (4)	
H4	0.5153	0.1071	0.3318	0.013*	
C5	0.2754 (4)	0.10781 (19)	0.4680 (2)	0.0106 (5)	
H5	0.2313	0.03	0.4606	0.013*	
C6	0.1737 (4)	0.18136 (19)	0.5564 (2)	0.0111 (5)	
H6	0.0632	0.1503	0.6089	0.013*	
C7	0.6781 (4)	0.33471 (19)	0.3358 (2)	0.0097 (4)	
Cu1	0.5	0.5	0.5	0.00820 (13)	
N1	0.2267 (3)	0.29375 (16)	0.5696 (2)	0.0101 (4)	
N2	0.3890 (3)	0.33637 (16)	0.49462 (18)	0.0084 (4)	
O1	0.7022 (3)	0.44303 (13)	0.36438 (16)	0.0100 (3)	
O2	0.7873 (3)	0.28108 (13)	0.25561 (17)	0.0135 (4)	
O1W	0.1555 (3)	0.53934 (14)	0.30161 (17)	0.0121 (3)	
H1WA	0.014 (3)	0.514 (2)	0.321 (3)	0.024 (8)*	
H1WB	0.149 (6)	0.6143 (9)	0.292 (4)	0.051 (11)*	

### Atomic displacement parameters (Å^2^)

**Table:**

	*U*^11^	*U*^22^	*U*^33^	*U*^12^	*U*^13^	*U*^23^
C3	0.0088 (10)	0.0095 (11)	0.0063 (10)	0.0001 (8)	0.0005 (8)	0.0003 (8)
C4	0.0128 (11)	0.0094 (11)	0.0094 (10)	0.0035 (9)	0.0014 (9)	−0.0004 (8)
C5	0.0107 (10)	0.0082 (11)	0.0117 (11)	−0.0007 (8)	−0.0007 (9)	0.0019 (8)
C6	0.0106 (10)	0.0125 (11)	0.0106 (11)	−0.0011 (9)	0.0028 (9)	0.0014 (9)
C7	0.0091 (10)	0.0120 (11)	0.0080 (10)	−0.0007 (8)	0.0018 (9)	0.0001 (9)
Cu1	0.0111 (2)	0.0052 (2)	0.0101 (2)	−0.00061 (14)	0.00618 (15)	−0.00061 (14)
N1	0.0107 (9)	0.0099 (9)	0.0104 (9)	−0.0019 (7)	0.0039 (8)	−0.0002 (7)
N2	0.0091 (9)	0.0092 (9)	0.0072 (9)	0.0007 (7)	0.0021 (7)	0.0013 (7)
O1	0.0112 (7)	0.0071 (8)	0.0128 (8)	−0.0012 (6)	0.0052 (6)	0.0004 (6)
O2	0.0158 (8)	0.0120 (8)	0.0152 (8)	−0.0009 (6)	0.0094 (7)	−0.0033 (6)
O1W	0.0101 (8)	0.0105 (8)	0.0161 (8)	0.0006 (6)	0.0037 (7)	0.0023 (7)

### Geometric parameters (Å, º)

**Table:**

C3—N2	1.334 (3)	C7—C3	1.520 (3)
C3—C4	1.395 (3)	Cu1—O1^i^	1.9792 (15)
C3—C7	1.520 (3)	Cu1—O1	1.9792 (15)
C4—C5	1.374 (3)	Cu1—N2	1.9822 (18)
C4—H4	0.93	Cu1—N2^i^	1.9822 (18)
C5—C6	1.393 (3)	Cu1—O1W	2.4207 (16)
C5—H5	0.93	Cu1—O1W^i^	2.4207 (16)
C6—N1	1.331 (3)	N1—N2	1.339 (3)
C6—H6	0.93	O1W—H1WA	0.872 (10)
C7—O2	1.231 (3)	O1W—H1WB	0.872 (10)
C7—O1	1.283 (3)		
			
N2—C3—C4	121.7 (2)	O1^i^—Cu1—N2^i^	82.52 (7)
N2—C3—C7	114.28 (19)	N2—Cu1—N2^i^	180
C4—C3—C7	124.0 (2)	O1—Cu1—O1W	88.90 (6)
C5—C4—C3	116.6 (2)	O1^i^—Cu1—O1W	91.10 (6)
C5—C4—H4	121.7	N2—Cu1—O1W	88.82 (6)
C3—C4—H4	121.7	N2^i^—Cu1—O1W	91.18 (6)
C4—C5—C6	118.6 (2)	O1—Cu1—O1W^i^	91.10 (6)
C4—C5—H5	120.7	O1^i^—Cu1—O1W^i^	88.90 (6)
C6—C5—H5	120.7	N2—Cu1—O1W^i^	91.18 (6)
N1—C6—C5	123.4 (2)	N2^i^—Cu1—O1W^i^	88.82 (6)
N1—C6—H6	118.3	O1W—Cu1—O1W^i^	180
C5—C6—H6	118.3	C6—N1—N2	117.37 (19)
O2—C7—O1	125.8 (2)	C3—N2—N1	122.33 (19)
O2—C7—C3	119.1 (2)	C3—N2—Cu1	113.24 (15)
O1—C7—C3	115.06 (19)	N1—N2—Cu1	124.43 (14)
O1^i^—Cu1—O1	180	C7—O1—Cu1	114.89 (13)
O1—Cu1—N2	82.52 (7)	Cu1—O1W—H1WA	109.8 (19)
O1^i^—Cu1—N2	97.48 (7)	Cu1—O1W—H1WB	106 (2)
O1—Cu1—N2^i^	97.48 (7)	H1WA—O1W—H1WB	109 (2)
			
N2—C3—C4—C5	−0.8 (3)	O1W—Cu1—N2—C3	89.46 (15)
C7—C3—C4—C5	179.22 (19)	O1W^i^—Cu1—N2—C3	−90.54 (15)
C3—C4—C5—C6	−0.2 (3)	O1—Cu1—N2—N1	179.32 (17)
C4—C5—C6—N1	1.1 (3)	O1^i^—Cu1—N2—N1	−0.68 (17)
C5—C6—N1—N2	−0.9 (3)	O1W—Cu1—N2—N1	−91.63 (16)
C4—C3—N2—N1	1.0 (3)	O1W^i^—Cu1—N2—N1	88.37 (16)
C7—C3—N2—N1	−179.04 (18)	O2—C7—O1—Cu1	−179.34 (18)
C4—C3—N2—Cu1	179.92 (16)	C3—C7—O1—Cu1	0.8 (2)
C7—C3—N2—Cu1	−0.1 (2)	N2—Cu1—O1^i^—C7^i^	−179.30 (15)
C6—N1—N2—C3	−0.1 (3)	N2—Cu1—O1—C7	−0.70 (15)
C6—N1—N2—Cu1	−178.91 (15)	O1W—Cu1—O1—C7	−89.65 (15)
O1—Cu1—N2—C3	0.41 (14)	O1W^i^—Cu1—O1—C7	90.35 (15)
O1^i^—Cu1—N2—C3	−179.59 (14)		

Symmetry code: (i) −*x*+1, −*y*+1, −*z*+1.

### Hydrogen-bond geometry (Å, º)

**Table:**

*D*—H···*A*	*D*—H	H···*A*	*D*···*A*	*D*—H···*A*
O1*W*—H1*WA*···O1^ii^	0.87 (2)	1.99 (2)	2.865 (2)	175 (2)
O1*W*—H1*WB*···O2^iii^	0.87 (1)	2.03 (2)	2.878 (2)	165 (3)
C4—H4···O1*W*^iv^	0.93	2.52	3.403 (3)	158
C6—H6···O2^v^	0.93	2.39	3.141 (3)	138

Symmetry codes: (ii) *x*−1, *y*, *z*; (iii) −*x*+1, *y*+1/2, −*z*+1/2; (iv) −*x*+1, *y*−1/2, −*z*+1/2; (v) *x*−1, −*y*+1/2, *z*+1/2.

## References

[bb1] Agilent (2012). *CrysAlis PRO* and *CrysAlis RED* Agilent Technologies Inc., Santa Clara, CA, USA.

[bb2] Artetxe, B., Reinoso, S., San Felices, L., Martín-Caballero, J. & Gutiérrez-Zorrilla, J. M. (2013). *Acta Cryst.* E**69**, m420–m421. 10.1107/S1600536813017340 PMC3772443 24046586

[bb3] Brandenburg, K. (2010). *DIAMOND* Crystal Impact GbR, Bonn, Germany.

[bb4] Dolomanov, O. V., Bourhis, L. J., Gildea, R. J., Howard, J. A. K. & Puschmann, H. (2009). *J. Appl. Cryst.* **42**, 339–341.

[bb5] Farrugia, L. J. (2012). *J. Appl. Cryst.* **45**, 849–854.

[bb6] Gryz, M., Starosta, W. & Leciejewicz, J. (2004). *Acta Cryst.* E**60**, m1481–m1483.

[bb7] Sheldrick, G. M. (2008). *Acta Cryst.* A**64**, 112–122. 10.1107/S0108767307043930 18156677

